# [3-Hy­droxy­methyl-1,4-bis­(4-methyl­phen­yl)naphthalen-2-yl]methanol

**DOI:** 10.1107/S160053681100986X

**Published:** 2011-03-19

**Authors:** P. Narayanan, K. Sethusankar, Meganathan Nandakumar, Arasambattu K. Mohanakrishnan

**Affiliations:** a Department of Physics, RKM Vivekananda College (Autonomous), Chennai 600 004, India; b Department of Organic Chemistry, University of Madras, Maraimalai Campus, Chennai 600 025, India

## Abstract

In the title compound, C_26_H_24_O_2_, the crowded naphthalene ring system is essentially planar [maximum deviation of 0.027 (2) Å for one of the C atoms of the unsubstituted ring]. In the crystal, mol­ecules are connected by O—H⋯O hydrogen bonds into chains along the *a* axis. Pairs of the oppositely oriented chains are further cross-linked by O—H⋯O hydrogen bonds, forming infinte bands of alternating *R*_4_^4^(8) dimers and *R*_2_^2^(14) motifs.

## Related literature

For applications of naphthalene derivatives, see: Fukuzumi *et al.* (1994 ▶); Tsukada *et al.* (1994 ▶). For related structures, see: Wang *et al.* (2008 ▶); Çelik *et al.* (2009 ▶). For graph-set notation, see: Bernstein *et al.* (1995 ▶). For asymmetry parameters, see: Nardelli (1983 ▶).
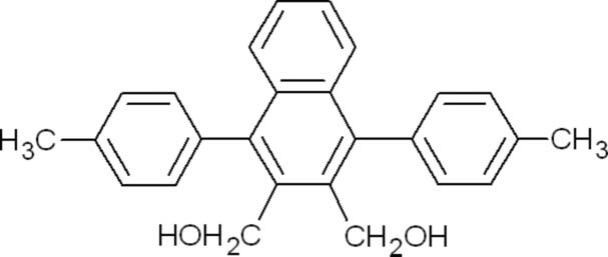


## Experimental

### 

#### Crystal data

C_26_H_24_O_2_
*M* *_r_* = 368.45
Triclinic, 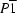

*a* = 6.3525 (3) Å
*b* = 10.0192 (4) Å
*c* = 16.4654 (7) Å
α = 77.723 (2)°
β = 81.870 (2)°
γ = 78.108 (2)°
*V* = 996.80 (7) Å^3^
*Z* = 2
Mo *K*α radiation
μ = 0.08 mm^−1^
*T* = 293 K
0.30 × 0.25 × 0.20 mm

#### Data collection

Bruker Kappa APEXII CCD diffractometer
19109 measured reflections
3910 independent reflections
3061 reflections with *I* > 2σ(*I*)
*R*_int_ = 0.036

#### Refinement

*R*[*F* ^2^ > 2σ(*F* ^2^)] = 0.045
*wR*(*F* ^2^) = 0.133
*S* = 1.04
3910 reflections
257 parameters
H-atom parameters constrained
Δρ_max_ = 0.22 e Å^−3^
Δρ_min_ = −0.19 e Å^−3^

### 

Data collection: *APEX2* (Bruker, 2004 ▶); cell refinement: *SAINT* (Bruker, 2004 ▶); data reduction: *SAINT*; program(s) used to solve structure: *SHELXS97* (Sheldrick, 2008 ▶); program(s) used to refine structure: *SHELXL97* (Sheldrick, 2008 ▶); molecular graphics: *ORTEP-3* (Farrugia, 1997 ▶); software used to prepare material for publication: *SHELXL97* and *PLATON* (Spek, 2009 ▶).

## Supplementary Material

Crystal structure: contains datablocks global, I. DOI: 10.1107/S160053681100986X/ld2002sup1.cif

Structure factors: contains datablocks I. DOI: 10.1107/S160053681100986X/ld2002Isup2.hkl

Additional supplementary materials:  crystallographic information; 3D view; checkCIF report

## Figures and Tables

**Table 1:** Hydrogen-bond geometry (Å, °)

*D*—H⋯*A*	*D*—H	H⋯*A*	*D*⋯*A*	*D*—H⋯*A*
O1—H1*A*⋯O2^i^	0.82	1.89	2.7065 (18)	175
O2—H2*A*⋯O1^ii^	0.82	2.02	2.7235 (17)	143

Symmetry codes: (i) 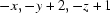; (ii) 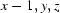.

**Fig. 1.**
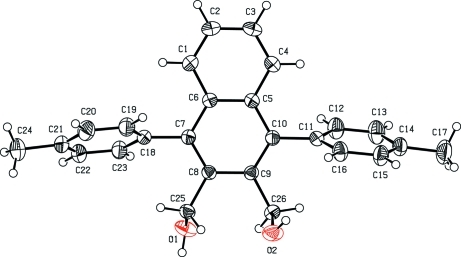
The molecular structure of the title compound with the atom numbering scheme. Displacement ellipsoids are drawn at 50% probability level. H atoms are presented as small spheres of arbitary radius.

**Fig. 2.**
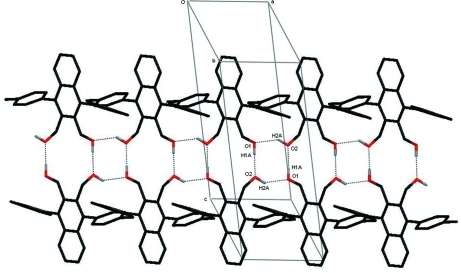
Part of crystal structure of the title compound, showing formation of the one-dimensional band along x axis with dimeric R44(8) and R22(14)grsaphset motifs.

## supplementary crystallographic information

### Comment

1,4-Naphthalene derivatives are important synthones in preparation of polymers (Fukuzumi *et al.*,1994; Tsukada *et al.*,1994). The title compound, C_26_H_24_O_2_, was synthesized from diethyl(1,4-di-*p*-tolylnaphthalene-2,3-dicarboxylate).

The naphthalene moiety is essentially planar with a maximum deviation of 0.027 (2) Å for atom C4. The X-ray study confirms the molecular structure and atom connectivity as shown in the Fig.1. The naphthalene moiety forms dihedral angles of 72.91 (7)° and 69.58 (7)° with two adjacent benzene rings C11···C16 and C18···C23, respectively. Atoms O1 and O2 are rotated in opposite directions from the naphthalene plane, deviating by -1.227 (1)Å and 1.218 (1) Å, repectively (Nardelli, 1983).

Hydrogen bonds O2—H2A···O1 connect adjacent molecules to form one-dimensional chains along crystallographic x axis. Pairs of the oppositely oriented chains are further cross-linked by O1—H1A···O2 hygrogen bonds forming infinite bands made of alternate *R*^4^_4_(8) dimers and *R*^2^_2_(14) graphset motifs (Bernstein *et al.* 1995) as shown in Fig.2.

### Experimental

LiAlH_4_ (2.77 g, 73.01 mmol) in THF (100 ml) was added to an oven-dried flask. Diethyl(1,4-di-*p*-tolylnaphthalene-2,3-dicarboxylate) (15 g, 33.18 mmol) was dissolved in anhydrous THF (100 ml) and added dropwise to the LiAlH_4_ solution. The reaction was stirred for 12 h at room temperature. The mixture was cooled to 273 K and quenched very slowly by addition of water (20 ml) and 10% HCl (20 ml). Dichloromethane (200 ml) was added to the mixture and then organic layer was separated and washed with water (2x100 ml). The solution was dried with Na_2_SO_4_ and organic solvent was evaporated to give crude product. The crude product was purified by column chromotography to give the title compound (12 g) in 90% yield as a white solid.

### Refinement

Positions of hydrogen atoms were localized from the difference electron density maps and their distances were geometrically constrained. The H atoms of hydroxy groups were constrained to a distance of d(O—H) = 0.82 Å with *U*iso(H) = 1.5*U*eq(O). The H atoms bound to the C atoms were treated as riding atoms, with d(C—H)=0.93 Å and *U*iso(H) = 1.2Ueq(C) for aromatic, d(C—H)=0.97 Å and *U*iso(H)=1.2Ueq(C) for methylene and d(C—H)=0.96 Å and *U*iso(H) =1.5*U*eq(C) for methyl groups. The rotation angles for hydroxy and methyl groups were optimized by least squares.

### Crystal data

**Table:**

C_26_H_24_O_2_	*Z* = 2
*M**_r_* = 368.45	*F*(000) = 392
Triclinic, *P*1	*D*_x_ = 1.228 Mg m^−^^3^
Hall symbol: -p 1	Mo *K*α radiation, λ = 0.71073 Å
*a* = 6.3525 (3) Å	Cell parameters from 3910 reflections
*b* = 10.0192 (4) Å	θ = 1.0–26.0°
*c* = 16.4654 (7) Å	µ = 0.08 mm^−^^1^
α = 77.723 (2)°	*T* = 293 K
β = 81.870 (2)°	Block, colorless
γ = 78.108 (2)°	0.30 × 0.25 × 0.20 mm
*V* = 996.80 (7) Å^3^	

### Data collection

**Table:**

Bruker Kappa APEXII CCD diffractometer	3061 reflections with *I* > 2σ(*I*)
Radiation source: fine-focus sealed tube	*R*_int_ = 0.036
graphite	θ_max_ = 26.0°, θ_min_ = 2.3°
ω scans	*h* = −7→7
19109 measured reflections	*k* = −12→12
3910 independent reflections	*l* = −20→20

### Refinement

**Table:**

Refinement on *F*^2^	Primary atom site location: structure-invariant direct methods
Least-squares matrix: full	Secondary atom site location: difference Fourier map
*R*[*F*^2^ > 2σ(*F*^2^)] = 0.045	Hydrogen site location: inferred from neighbouring sites
*wR*(*F*^2^) = 0.133	H-atom parameters constrained
*S* = 1.04	*w* = 1/[σ^2^(*F*_o_^2^) + (0.0683*P*)^2^ + 0.2321*P*] where *P* = (*F*_o_^2^ + 2*F*_c_^2^)/3
3910 reflections	(Δ/σ)_max_ = 0.024
257 parameters	Δρ_max_ = 0.22 e Å^−^^3^
0 restraints	Δρ_min_ = −0.19 e Å^−^^3^

### Special details

**Table:**

Geometry. All e.s.d.'s (except the e.s.d. in the dihedral angle between two l.s. planes) are estimated using the full covariance matrix. The cell e.s.d.'s are taken into account individually in the estimation of e.s.d.'s in distances, angles and torsion angles; correlations between e.s.d.'s in cell parameters are only used when they are defined by crystal symmetry. An approximate (isotropic) treatment of cell e.s.d.'s is used for estimating e.s.d.'s involving l.s. planes.
Refinement. Refinement of *F*^2^ against ALL reflections. The weighted *R*-factor *wR* and goodness of fit *S* are based on *F*^2^, conventional *R*-factors *R* are based on *F*, with *F* set to zero for negative *F*^2^. The threshold expression of *F*^2^ > σ(*F*^2^) is used only for calculating *R*-factors(gt) *etc*. and is not relevant to the choice of reflections for refinement. *R*-factors based on *F*^2^ are statistically about twice as large as those based on *F*, and *R*- factors based on ALL data will be even larger.

### Fractional atomic coordinates and isotropic or equivalent isotropic displacement parameters (Å^2^)

**Table:**

	*x*	*y*	*z*	*U*_iso_*/*U*_eq_	
C1	0.2706 (3)	0.94217 (18)	0.07678 (10)	0.0492 (4)	
H1	0.3534	1.0114	0.0585	0.059*	
C2	0.2351 (3)	0.8678 (2)	0.02158 (11)	0.0587 (5)	
H2	0.2943	0.8860	−0.0337	0.070*	
C3	0.1105 (3)	0.76456 (19)	0.04756 (11)	0.0573 (5)	
H3	0.0849	0.7150	0.0093	0.069*	
C4	0.0258 (3)	0.73548 (17)	0.12864 (10)	0.0479 (4)	
H4	−0.0578	0.6664	0.1450	0.057*	
C5	0.0626 (2)	0.80827 (14)	0.18835 (9)	0.0355 (3)	
C6	0.1845 (2)	0.91646 (15)	0.16136 (9)	0.0361 (3)	
C7	0.2177 (2)	0.99509 (14)	0.22017 (9)	0.0329 (3)	
C8	0.1344 (2)	0.96324 (13)	0.30250 (8)	0.0307 (3)	
C9	0.0165 (2)	0.85221 (13)	0.32980 (8)	0.0309 (3)	
C10	−0.0209 (2)	0.77660 (14)	0.27435 (9)	0.0327 (3)	
C11	−0.1450 (2)	0.66107 (14)	0.30361 (9)	0.0345 (3)	
C12	−0.0415 (3)	0.52369 (16)	0.31375 (12)	0.0505 (4)	
H12	0.1059	0.5028	0.2974	0.061*	
C13	−0.1532 (3)	0.41753 (16)	0.34770 (13)	0.0577 (5)	
H13	−0.0792	0.3262	0.3538	0.069*	
C14	−0.3727 (3)	0.44283 (16)	0.37296 (10)	0.0467 (4)	
C15	−0.4757 (3)	0.57913 (17)	0.36041 (11)	0.0500 (4)	
H15	−0.6238	0.5997	0.3755	0.060*	
C16	−0.3654 (3)	0.68645 (15)	0.32604 (11)	0.0451 (4)	
H16	−0.4408	0.7774	0.3179	0.054*	
C17	−0.4920 (4)	0.3264 (2)	0.41238 (15)	0.0716 (6)	
H17A	−0.5038	0.2747	0.3709	0.107*	
H17B	−0.6340	0.3640	0.4352	0.107*	
H17C	−0.4144	0.2660	0.4562	0.107*	
C18	0.3357 (2)	1.11445 (14)	0.19167 (9)	0.0355 (3)	
C19	0.2405 (3)	1.23562 (16)	0.14231 (10)	0.0469 (4)	
H19	0.1093	1.2391	0.1223	0.056*	
C20	0.3375 (3)	1.35125 (17)	0.12230 (11)	0.0536 (4)	
H20	0.2695	1.4316	0.0894	0.064*	
C21	0.5335 (3)	1.35040 (17)	0.15006 (11)	0.0492 (4)	
C22	0.6302 (3)	1.22817 (17)	0.19724 (11)	0.0486 (4)	
H22	0.7634	1.2239	0.2158	0.058*	
C23	0.5348 (3)	1.11207 (16)	0.21764 (10)	0.0428 (4)	
H23	0.6050	1.0311	0.2492	0.051*	
C24	0.6348 (4)	1.4786 (2)	0.13058 (14)	0.0717 (6)	
H24A	0.6240	1.5217	0.0730	0.108*	
H24B	0.7843	1.4531	0.1407	0.108*	
H24C	0.5605	1.5426	0.1656	0.108*	
C25	0.1635 (2)	1.04605 (14)	0.36509 (9)	0.0357 (3)	
H25A	0.2066	1.1326	0.3360	0.043*	
H25B	0.0275	1.0681	0.3987	0.043*	
C26	−0.0681 (3)	0.82015 (16)	0.42085 (9)	0.0400 (4)	
H26A	−0.1094	0.7294	0.4332	0.048*	
H26B	0.0461	0.8167	0.4550	0.048*	
O1	0.32387 (18)	0.96912 (12)	0.41770 (8)	0.0525 (3)	
H1A	0.3068	0.9989	0.4613	0.074 (7)*	
O2	−0.24973 (19)	0.92102 (14)	0.44206 (8)	0.0564 (3)	
H2A	−0.3508	0.9189	0.4167	0.068 (7)*	

### Atomic displacement parameters (Å^2^)

**Table:**

	*U*^11^	*U*^22^	*U*^33^	*U*^12^	*U*^13^	*U*^23^
C1	0.0593 (10)	0.0559 (10)	0.0370 (9)	−0.0265 (8)	0.0082 (7)	−0.0120 (7)
C2	0.0800 (13)	0.0701 (12)	0.0324 (9)	−0.0304 (10)	0.0092 (8)	−0.0175 (8)
C3	0.0814 (13)	0.0635 (11)	0.0382 (9)	−0.0269 (10)	−0.0022 (9)	−0.0232 (8)
C4	0.0620 (11)	0.0479 (9)	0.0418 (9)	−0.0244 (8)	−0.0025 (8)	−0.0143 (7)
C5	0.0381 (8)	0.0364 (7)	0.0344 (8)	−0.0103 (6)	−0.0012 (6)	−0.0105 (6)
C6	0.0381 (8)	0.0397 (8)	0.0328 (8)	−0.0121 (6)	0.0008 (6)	−0.0097 (6)
C7	0.0320 (7)	0.0340 (7)	0.0335 (7)	−0.0085 (6)	−0.0014 (6)	−0.0071 (6)
C8	0.0286 (7)	0.0313 (7)	0.0328 (7)	−0.0045 (5)	−0.0024 (6)	−0.0092 (5)
C9	0.0287 (7)	0.0327 (7)	0.0308 (7)	−0.0051 (5)	−0.0012 (5)	−0.0068 (5)
C10	0.0326 (7)	0.0321 (7)	0.0337 (8)	−0.0073 (6)	−0.0020 (6)	−0.0066 (6)
C11	0.0386 (8)	0.0329 (7)	0.0340 (7)	−0.0100 (6)	−0.0019 (6)	−0.0082 (6)
C12	0.0408 (9)	0.0375 (8)	0.0711 (12)	−0.0061 (7)	0.0005 (8)	−0.0105 (8)
C13	0.0568 (11)	0.0297 (8)	0.0824 (13)	−0.0062 (7)	−0.0069 (10)	−0.0029 (8)
C14	0.0551 (10)	0.0410 (8)	0.0464 (9)	−0.0211 (7)	−0.0053 (8)	−0.0011 (7)
C15	0.0389 (9)	0.0485 (9)	0.0636 (11)	−0.0159 (7)	0.0032 (8)	−0.0104 (8)
C16	0.0407 (9)	0.0328 (7)	0.0608 (10)	−0.0076 (6)	−0.0019 (7)	−0.0078 (7)
C17	0.0820 (14)	0.0565 (11)	0.0770 (14)	−0.0367 (11)	−0.0019 (11)	0.0050 (10)
C18	0.0405 (8)	0.0360 (7)	0.0322 (7)	−0.0132 (6)	0.0020 (6)	−0.0087 (6)
C19	0.0477 (9)	0.0483 (9)	0.0458 (9)	−0.0163 (7)	−0.0084 (7)	−0.0019 (7)
C20	0.0650 (11)	0.0402 (8)	0.0524 (10)	−0.0143 (8)	−0.0065 (9)	0.0030 (7)
C21	0.0617 (11)	0.0452 (9)	0.0454 (9)	−0.0260 (8)	0.0069 (8)	−0.0115 (7)
C22	0.0474 (9)	0.0536 (9)	0.0504 (10)	−0.0232 (8)	−0.0038 (8)	−0.0095 (8)
C23	0.0420 (8)	0.0414 (8)	0.0455 (9)	−0.0136 (7)	−0.0047 (7)	−0.0035 (7)
C24	0.0944 (16)	0.0555 (11)	0.0736 (14)	−0.0427 (11)	0.0022 (12)	−0.0094 (10)
C25	0.0356 (8)	0.0363 (7)	0.0372 (8)	−0.0065 (6)	−0.0008 (6)	−0.0134 (6)
C26	0.0411 (8)	0.0482 (8)	0.0328 (8)	−0.0151 (7)	0.0018 (6)	−0.0092 (6)
O1	0.0477 (7)	0.0649 (7)	0.0515 (7)	0.0002 (6)	−0.0165 (5)	−0.0285 (6)
O2	0.0388 (6)	0.0868 (9)	0.0503 (7)	−0.0135 (6)	0.0076 (5)	−0.0337 (6)

### Geometric parameters (Å, °)

**Table:**

C1—C2	1.360 (2)	C15—H15	0.9300
C1—C6	1.413 (2)	C16—H16	0.9300
C1—H1	0.9300	C17—H17A	0.9600
C2—C3	1.390 (2)	C17—H17B	0.9600
C2—H2	0.9300	C17—H17C	0.9600
C3—C4	1.361 (2)	C18—C19	1.384 (2)
C3—H3	0.9300	C18—C23	1.386 (2)
C4—C5	1.412 (2)	C19—C20	1.380 (2)
C4—H4	0.9300	C19—H19	0.9300
C5—C6	1.4198 (19)	C20—C21	1.384 (3)
C5—C10	1.433 (2)	C20—H20	0.9300
C6—C7	1.4333 (19)	C21—C22	1.379 (2)
C7—C8	1.379 (2)	C21—C24	1.508 (2)
C7—C18	1.4976 (18)	C22—C23	1.378 (2)
C8—C9	1.4266 (18)	C22—H22	0.9300
C8—C25	1.5052 (18)	C23—H23	0.9300
C9—C10	1.3762 (19)	C24—H24A	0.9600
C9—C26	1.509 (2)	C24—H24B	0.9600
C10—C11	1.4916 (19)	C24—H24C	0.9600
C11—C16	1.381 (2)	C25—O1	1.4228 (18)
C11—C12	1.384 (2)	C25—H25A	0.9700
C12—C13	1.376 (2)	C25—H25B	0.9700
C12—H12	0.9300	C26—O2	1.425 (2)
C13—C14	1.384 (3)	C26—H26A	0.9700
C13—H13	0.9300	C26—H26B	0.9700
C14—C15	1.373 (2)	O1—H1A	0.8200
C14—C17	1.503 (2)	O2—H2A	0.8200
C15—C16	1.381 (2)		
			
C2—C1—C6	121.35 (15)	C11—C16—H16	119.4
C2—C1—H1	119.3	C15—C16—H16	119.4
C6—C1—H1	119.3	C14—C17—H17A	109.5
C1—C2—C3	120.19 (16)	C14—C17—H17B	109.5
C1—C2—H2	119.9	H17A—C17—H17B	109.5
C3—C2—H2	119.9	C14—C17—H17C	109.5
C4—C3—C2	120.44 (15)	H17A—C17—H17C	109.5
C4—C3—H3	119.8	H17B—C17—H17C	109.5
C2—C3—H3	119.8	C19—C18—C23	117.61 (13)
C3—C4—C5	121.18 (15)	C19—C18—C7	120.27 (13)
C3—C4—H4	119.4	C23—C18—C7	121.98 (13)
C5—C4—H4	119.4	C20—C19—C18	121.00 (15)
C4—C5—C6	118.41 (13)	C20—C19—H19	119.5
C4—C5—C10	121.88 (13)	C18—C19—H19	119.5
C6—C5—C10	119.71 (12)	C19—C20—C21	121.50 (15)
C1—C6—C5	118.38 (13)	C19—C20—H20	119.2
C1—C6—C7	122.13 (13)	C21—C20—H20	119.2
C5—C6—C7	119.49 (13)	C22—C21—C20	117.16 (14)
C8—C7—C6	119.68 (12)	C22—C21—C24	121.58 (17)
C8—C7—C18	120.20 (12)	C20—C21—C24	121.26 (17)
C6—C7—C18	120.08 (12)	C23—C22—C21	121.83 (16)
C7—C8—C9	120.66 (12)	C23—C22—H22	119.1
C7—C8—C25	120.67 (12)	C21—C22—H22	119.1
C9—C8—C25	118.67 (12)	C22—C23—C18	120.85 (15)
C10—C9—C8	120.84 (12)	C22—C23—H23	119.6
C10—C9—C26	120.62 (12)	C18—C23—H23	119.6
C8—C9—C26	118.53 (12)	C21—C24—H24A	109.5
C9—C10—C5	119.59 (12)	C21—C24—H24B	109.5
C9—C10—C11	120.34 (12)	H24A—C24—H24B	109.5
C5—C10—C11	120.06 (12)	C21—C24—H24C	109.5
C16—C11—C12	117.24 (13)	H24A—C24—H24C	109.5
C16—C11—C10	121.55 (12)	H24B—C24—H24C	109.5
C12—C11—C10	121.11 (13)	O1—C25—C8	110.26 (11)
C13—C12—C11	121.04 (15)	O1—C25—H25A	109.6
C13—C12—H12	119.5	C8—C25—H25A	109.6
C11—C12—H12	119.5	O1—C25—H25B	109.6
C12—C13—C14	121.84 (15)	C8—C25—H25B	109.6
C12—C13—H13	119.1	H25A—C25—H25B	108.1
C14—C13—H13	119.1	O2—C26—C9	112.15 (12)
C15—C14—C13	116.83 (14)	O2—C26—H26A	109.2
C15—C14—C17	121.72 (17)	C9—C26—H26A	109.2
C13—C14—C17	121.45 (16)	O2—C26—H26B	109.2
C14—C15—C16	121.78 (15)	C9—C26—H26B	109.2
C14—C15—H15	119.1	H26A—C26—H26B	107.9
C16—C15—H15	119.1	C25—O1—H1A	109.5
C11—C16—C15	121.19 (14)	C26—O2—H2A	109.5
			
C6—C1—C2—C3	0.5 (3)	C5—C10—C11—C16	−108.92 (17)
C1—C2—C3—C4	−1.0 (3)	C9—C10—C11—C12	−104.47 (17)
C2—C3—C4—C5	−0.3 (3)	C5—C10—C11—C12	74.69 (19)
C3—C4—C5—C6	2.1 (3)	C16—C11—C12—C13	−2.3 (3)
C3—C4—C5—C10	−178.42 (16)	C10—C11—C12—C13	174.20 (16)
C2—C1—C6—C5	1.3 (3)	C11—C12—C13—C14	0.1 (3)
C2—C1—C6—C7	−179.13 (16)	C12—C13—C14—C15	1.8 (3)
C4—C5—C6—C1	−2.6 (2)	C12—C13—C14—C17	−178.12 (18)
C10—C5—C6—C1	177.94 (14)	C13—C14—C15—C16	−1.5 (3)
C4—C5—C6—C7	177.87 (14)	C17—C14—C15—C16	178.43 (17)
C10—C5—C6—C7	−1.6 (2)	C12—C11—C16—C15	2.6 (2)
C1—C6—C7—C8	−178.39 (14)	C10—C11—C16—C15	−173.88 (15)
C5—C6—C7—C8	1.1 (2)	C14—C15—C16—C11	−0.8 (3)
C1—C6—C7—C18	3.8 (2)	C8—C7—C18—C19	−107.46 (17)
C5—C6—C7—C18	−176.70 (13)	C6—C7—C18—C19	70.37 (19)
C6—C7—C8—C9	0.4 (2)	C8—C7—C18—C23	68.19 (19)
C18—C7—C8—C9	178.24 (12)	C6—C7—C18—C23	−113.98 (16)
C6—C7—C8—C25	−178.83 (12)	C23—C18—C19—C20	−2.2 (2)
C18—C7—C8—C25	−1.0 (2)	C7—C18—C19—C20	173.59 (15)
C7—C8—C9—C10	−1.5 (2)	C18—C19—C20—C21	0.5 (3)
C25—C8—C9—C10	177.73 (12)	C19—C20—C21—C22	1.2 (3)
C7—C8—C9—C26	179.01 (13)	C19—C20—C21—C24	−177.79 (17)
C25—C8—C9—C26	−1.75 (18)	C20—C21—C22—C23	−1.3 (3)
C8—C9—C10—C5	1.0 (2)	C24—C21—C22—C23	177.75 (17)
C26—C9—C10—C5	−179.50 (13)	C21—C22—C23—C18	−0.5 (3)
C8—C9—C10—C11	−179.79 (12)	C19—C18—C23—C22	2.2 (2)
C26—C9—C10—C11	−0.3 (2)	C7—C18—C23—C22	−173.55 (14)
C4—C5—C10—C9	−178.93 (14)	C7—C8—C25—O1	−104.66 (15)
C6—C5—C10—C9	0.5 (2)	C9—C8—C25—O1	76.09 (16)
C4—C5—C10—C11	1.9 (2)	C10—C9—C26—O2	−105.53 (15)
C6—C5—C10—C11	−178.65 (13)	C8—C9—C26—O2	73.95 (16)
C9—C10—C11—C16	71.91 (19)		

### Hydrogen-bond geometry (Å, °)

**Table:**

*D*—H···*A*	*D*—H	H···*A*	*D*···*A*	*D*—H···*A*
O1—H1A···O2^i^	0.82	1.89	2.7065 (18)	175
O2—H2A···O1^ii^	0.82	2.02	2.7235 (17)	143

Symmetry codes: (i) −*x*, −*y*+2, −*z*+1; (ii) *x*−1, *y*, *z*.
